# Using Rosemary Essential Oil as a Potential Natural Preservative during Stirred-like Yogurt Making

**DOI:** 10.3390/foods11141993

**Published:** 2022-07-06

**Authors:** Dalia Gamal Kamel, Ali I. A. Mansour, Mohamed A. H. Nagm El-diin, Ahmed R. A. Hammam, Dipakkumar Mehta, Asmaa Mohamed Abdel-Rahman

**Affiliations:** 1 Dairy Science Department, Faculty of Agriculture, Assiut University, Assiut 71526, Egypt; ahmed.hammam@jacks.sdstate.edu; 2 Dairy Science Department, Faculty of Agriculture, Al-Azhar University, Assiut 71526, Egypt; alibahbo58@gmail.com (A.I.A.M.); Alkhdrawy65@gmail.com (M.A.H.N.E.-d.); 3 Dairy and Food Science Department, South Dakota State University, Brookings, SD 57006, USA; 4 Research and Development, Wells Enterprises Inc., Le Mars, IA 51031, USA; dsmehta@bluebunny.com; 5 Food Science and Technology Department, Faculty of Agriculture, Assiut University, Assiut 71526, Egypt

**Keywords:** stirred-like yogurt, rosemary essential oil, GC-MS, antimicrobial activity, acidity, lactic acid bacteria

## Abstract

The popularity of rosemary has grown as a natural alternative over the synthetic supplements due to its potential health benefits. The rosemary plant has been utilized to preserve food due to its ability to prevent oxidation and microbial contamination. The reason for this study was to determine the phytochemical components and antimicrobial activity of rosemary essential oil (REO) and the effect of REO addition (0.5 and 0.7%) on the chemical, microbiological, and sensory properties of stirred-like yogurt (SLY) during 16 days of storage at 4 °C. The obtained data observed that REO exhibited antimicrobial action against *Escherichia coli*, *Staphylococcus aureus*, and *Salmonella marcescens,* as well as fungi (*Aspergillus flavus*) and yeasts (*Candida albicans*). Increased REO to 0.7% accelerated (*p* < 0.05) the development of lactic acid bacteria (LAB) in SLY (8.3 log cfu/g) and delayed yeast growth up to 12 days. Molds and coliforms were also not found in the SLY samples with REO. In comparison to control samples, sensory results showed that the addition of REO improves the overall acceptance of SLY (*p* < 0.05). In conclusion, the current study found that REO could be used as a natural preservative during the production of SLY to extend shelf-life and promote LAB development.

## 1. Introduction

Consumers are generally aware of healthful, nutritious food products with a longer shelf-life. In food products, microbiological, enzymatic, physical, and chemical changes occur, resulting in a loss of quality, nutritional value, and safety [1]. Using synthetic preservatives, such as benzoic acid, to prevent food deterioration results in a kerosene-like odor [2]. As a result, the necessity for natural plant-derived antimicrobial activity (Bio-preservatives) to replace these artificial preservatives has grown [3,4]. Natural antioxidant ingredients were added to dairy products to boost antioxidant activity and anti-inflammatory characteristics, as well as providing a variety of health benefits [5]. In fact, essential oils have antimicrobial properties against yeasts, molds, and bacteria [3,6].

Natural phenolic compounds that have been found in plants and vegetables may decrease the risk of some diseases because of their antioxidant and free radical inhibition potentials imparted by the benzene ring and the hydroxyl group in their structures [7,8]. Rosemary (*Rosmarinus officialism* L.) oil is used as a food seasoning for food [9]. Rosemary essential oil (REO) has traditionally and largely been used as a medicinal herb, with a number of properties, such as anti-inflammatory, analgesic, astringent, antimicrobial, anti-rheumatic, carminative, antifungal, and antioxidant [10]. Yogurt is a coagulated milk product obtained by lactic acid fermentation through the action of *Lactobacillus bulgaricus* and *Streptococcus thermophilus* and is a popular product throughout the world [11]. The highest production or consumption of yogurt is in Mediterranean and Asian countries and in central Europe [12]. Low-calorie skimmed or half-skimmed yogurts have won popularity during the last decade [13]. It has been known for its nutraceutical, therapeutic, and probiotic effects, such as digestion enhancement, immune system boosting, anticarcinogenic activity, and reduction in serum cholesterol [13,14].

REO is used to improve the quality of yogurt [15]. Additionally, REO possesses potent antioxidant, antibacterial, and antimutagenic effects, as well as a distinct flavor [16]. Due to the strong odor of essential oils, their usage in foods has been limited [17]. As a result, the aim of this study was to assess the antioxidant properties and antimicrobial activity of REO, as well as examine two percentages of REO as a natural preservative during the production of stirred-like yogurt (SLY) and assess its effect on sensorial attributes, and chemical and microbiological changes, which occur during 16 days of storage.

## 2. Materials and Methods

### 2.1. Materials

The rosemary plants (*Rosmarinus officinalis* L.) were taken from the Floriculture Farm in November 2020 (Faculty of Agriculture, Assiut University, Assiut, Egypt). Fresh buffalo milk (6% fat) was obtained from the Animal Production Farm (Faculty of Agriculture, Assiut University, Assiut, Egypt). The Egyptian Microbial Cultures Collection (EMCC) at Cairo Microbiological Resources Center (Cairo MIRCEN), Faculty of Agriculture, Ain Shams University, Cairo, Egypt, provided *Lactococcus lactis* ss. *Lactis* ATCC 11454 and *Lactococcus lactis* ss. *cremoris* ATCC 19257.

### 2.2. Preparation of Samples

#### Extraction of Essential Oil

Essential oil was obtained by the method described by Abdel-Hameed et al. (10). A laboratory hot plate (Fisher Scientific, 50 Hz, MA 02454, USA), a five-liter flat-bottom conical flask, and a Clevenger system as condenser and oil collector were used to extract REO. In the container, 500 g of fresh cut leaves was immersed in 3 L of distilled water. Extraction took about 150 min at 100 °C until the essential oil stopped flowing. The essential oil was collected at the end of the experiment and dried over sodium sulphate before being filtered. The oil was kept in a brown glass vial at −20 °C until the chemical and biological tests were completed.

### 2.3. Manufacturing of SLY

**Figure 1:**
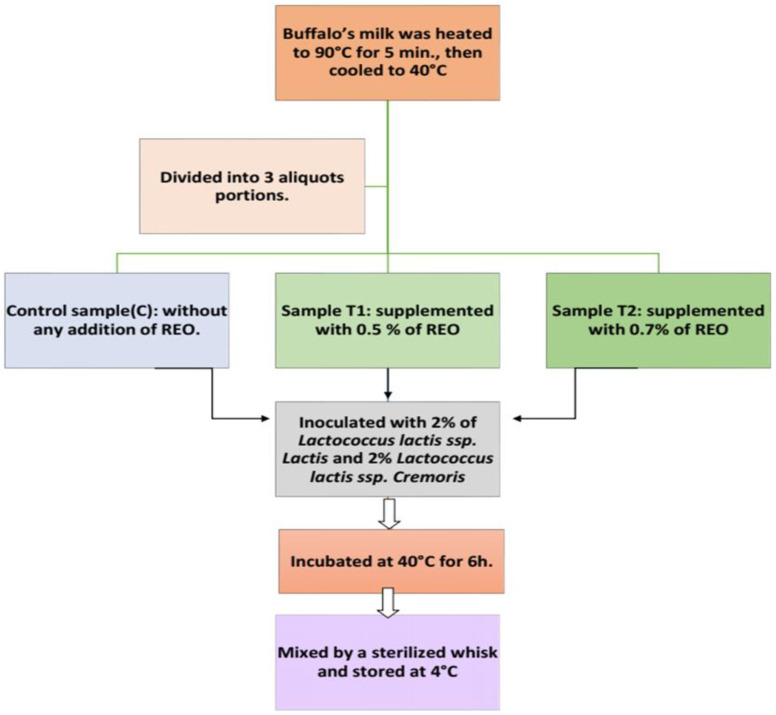
Flow sheet diagram for making SLY supplemented with 0.5 and 0.7% of REO.

Buffalo milk (6% fat) was heated at 90 °C for 5 min before being cooled to 40 °C. Three equal quantities of milk were divided. The first portion was used as a control (C) without REO addition, while samples T1 and T2 were supplemented with 0.5 and 0.7% (*v*/*w*) of REO, respectively. As such, 2% of *Lactococcus lactis* ss. Lactis (6.47 log cfu/mL of milk) and 2% *Lactococcus lactis* ss. *cremoris* (6.54 log cfu/mL of milk) were used as a starter culture for all treatments. After inoculation, the samples were mixed and incubated at 40 °C for 6 h. The SLY samples were then mixed with a sterile whisk and kept at 4 °C for 16 days (Figure 1). This experiment was carried out three times with three distinct batches of raw milk.

### 2.4. Proximate Composition Analysis

#### 2.4.1. Chemical Composition

Titratable acidity was determined (as lactic acid) according to Sadler and Murphy [18]. Total solids (TS) and total protein contents (TP) were determined according to the AOAC guidelines [19]. Fat content was determined by Gerber method according to Kleyn et al. [20].

#### 2.4.2. Determination of Total Phenolic Compounds

Total phenolics content was determined using the method of Singleton et al., and the results are reported in mg of gallic acid equivalents (mg GAE/100 g sample) [21].

#### 2.4.3. Determination of Total Flavonoids

The flavonoid content was measured as aluminum chloride colorimetric according to Marinova et al., and the results are represented as mg catechin equivalents (mg catechin/100 g sample) [22].

#### 2.4.4. Gas Chromatography-Mass Spectrometry (GC-MS) Analysis

The GC-MS analysis of the essential oil samples was carried out at the Department of Analytical Chemistry, Faculty of Science, Assiut University, to determine the volatile and semi-volatile chemicals. The extract of rosemary was made with isopropanol and ethyl acetate. The GC-MS analysis was performed with a Thermo Scientific TM TRACE 1300 coupled to an ISQ-7000 and a Thermo Scientific TM TG-6MB 5 ms (30 m×0.250 mm×1.00 m) column from Thermo Scientific. The temperature in the GC oven was kept at 100 °C for 15 min, then increased to 150 °C at a rate of 10 °C/min, and then to 200 °C at a rate of 5 °C/min. Furthermore, the temperature was raised to 250 °C at a rate of 5 °C per minute, and then to 280 °C at a rate of 5 °C per minute. Helium gas was used as the carrier, with a flow rate of 0.5 mL/min. The mass spectrometer was set to electron ionization mode, with a temperature of 320 °C for the ion source and 280 °C for the MS transfer line. The NIST 17 mass spectrum library (mainlib) was used to identify volatiles, and the results are represented as a percentage of the total GC area.

### 2.5. Microbiological Analysis

#### 2.5.1. Antimicrobial Activity of REO

The antimicrobial activity of REO was investigated against *Escherichia coli* (AUMC No. B-53), *Salmonella marcescens* (AUMC No. B-), *Staphylococcus aureus* (AUMC No. B-54), *Aspergillus flavus* (AUMC No. B-54), and *Candida albicans* (AUMC No. B-5). All the strains were obtained from Mycological Center, Faculty of Science, Assiut University, Egypt.

To prepare inoculum for bioassay, bacterial strains were individually cultured for 48 h in 100 mL conical flasks containing 30 mL nutrient broth medium. Fungi were grown for 7 days in 100 mL conical containing 30 mL Sabouraud’s dextrose broth. Nutrient agar and Sabouraud’s dextrose agar were, respectively, used for bacteria and fungi. After solidification of the media, 5 mm diameter cavities were cut in the solidified agar (4 cavities/plate) using sterile cork borer. After that REO was added (50 μL/cavity). Cultures were then incubated at 28 °C for 48 h in case of bacteria and up to 7 days in case of fungi. Results were read as the diameter (in mm) of inhibition zone around cavities.

- Minimum inhibitory concentrations (MICs):

To determine the minimum inhibitory concentrations (MICs), REO was diluted with dimethyl sulfoxide (DMSO) to prepare a series of concentrations (12.5, 25, 50 and 100%), which were pipetted into the cavities (50 μL/cavity) and similarly assayed as mentioned before and the least concentration (below which no activity) was recorded as the MIC.

#### 2.5.2. Standard Plate Count Technique

Fresh and after 4, 8, 12, and 16 days, the microbiological analysis was performed by using the usual plate count procedure, a total bacterial count (TBC) was plated on nutrient agar medium, and enumeration was completed [23]; before microbiological enumeration, the plates were incubated at 32 °C for 48–72 h. The MRS agar medium was used to obtain lactic acid bacterial (LAB) counts, and plates were incubated at 37 °C for 48 h [24]. In MacConkey broth media, coliform bacteria were discovered, and tubes were cultured for 24 h at 32 ± 1 °C [25]. Yeasts were counted on malt extract agar medium [26], whereas mold counts were counted on potato dextrose agar media, and the plates were cultured for 5 days at 25 ± 1 °C [23].

### 2.6. Sensory Evaluation

Sensory analysis of SLY samples was delineated by Kamel et al.’s guidelines with certain adjustments [27]. Flavor (45 points), body and texture (40 points), color and appearance (15 points), and overall acceptability (100 points) were all tested fresh and after 4, 8, 12, and 16 days of storage.

### 2.7. Statistical Analysis

The influence of REO addition, time, or their interaction on the features of SLY samples was explored statistically using R software (R x 643.3.3, Vienna, Austria) by ANOVA, testing a GLM for each variable. When significant differences were discovered at *p* < 0.05, the least significant difference (LSD) comparison test was used to separate the means.

## 3. Results

### 3.1. Phytochemical Components in REO

Total phenolics and total flavonoids: The total phenolic content in REO was recorded as 203.6 mg/kg, whereas total flavonoids concentration in our study was 488.98 mg catechin/100 g sample.

The chemical analysis and identification of REO by GC/MS: REO was analyzed and identified by using gas chromatography coupled with mass spectrometry (GC/MS). The obtained data are plotted in Table 1. From these data, it could be noticed that 91 compounds were identified. The concentrations of these compounds are varied between 0.0006 and 21.8229%. The most abundant components in REO are Bicyclo(2.2.1)heptan-2-one, 1,7,7-tri methyl-, (1R)-(camphor) (21.8229%), followed by α-Pinene (15.3175%), Caryophyllene (9.8533%), and Eucalyptol (9.3686%). Moderate amounts were found of Camphene (8.3843%), followed by Borneol (8.1423%), 3-Carene (8.2993%), and 3-Cyclohexene-1-methanol, à,à4-tri methyl (4.4136%). The minor components are Retinol (0.0006%) followed by Cinnamic acid, 4-hydroxy-3-methoxy- (0.0007%), and 4aà,4bá-Gibbane-1à,10á-dicarboxylic acid (0.0007%).

### 3.2. Antimicrobial Activity of REO

#### Minimum Inhibitory Concentration (MICs):

The diameter of inhibition zones was measured and taken as an indicator of the antimicrobial effect, as shown in Table 2. The range of inhibition zone diameter was varied from 0.0 to 14 mm in the studied bacteria. The MICs of REO were tested against Escherichia coli, Salmonella marcescens, and Staphylococcus aureus, and we found that adding 25% of REO gave the lowest inhibitory effect, with a 6 mm diameter inhibition zone. For 50% of REO extracts, the zones ranged from 7 to 8 mm. The data in the same table also indicate that the inhibition zone for REO (100% concentration) recorded 7 and 9 mm against Aspergillus flavus and Candida albicans, respectively.

### 3.3. The Effect of REO Addition on the Shelf-Life of SLY

#### 3.3.1. Gas Chromatography-Mass Spectrometry (GC-MS) Analysis

The data presented in Table 3 show the GC-MS analysis for SLY samples without and with REO addition. The data showed that some compounds in control samples (C), such as Geranyl isovalerate, Octadecane, 3-ethyl-5-(2-ethylbutyl), Phenol, 2,4-bis (1,1-di methyl ethyl), 1-Nonadecene, 7-Methyl-Z-tetradecen-1-ol acetate, Phthalic acid, butyl undecyl ester, Octadecanoic acid,(2-phenyl-1,3-dioxolan-4-yl) methyl ester, cis-, Tetradecanoic acid,3,3a,4,6a,7,8,9,10,10a, Estra-1,3,5(10)-trien-17á-ol, Ethanol, 2-(octadecyloxy)-, and 2-(16-Acetoxy-11-hydroxy-4,8,10,14-tetra methyl-3 decreased with storage, whereas Oleic acid, eicosyl ester, Phorbol, Pregn-5-ene-3, 11-dione and Acetic acid, and 17-(4-hydroxy-5-methoxy-1, 5-di methyl hexyl)-4, 4, 10, 13, 14-penta methyl increased with storage. Besides, other compounds appeared in control samples after storage for 16 days, including psi. psi.-Carotene,3,4-didehydro-1,2- dihydro-1-methoxy, Bis (benzimidazol-2-yl methyl) sulfone, Cholest-22-ene-21-ol, Bacteriochlorophyll-c-stearyl, Methyl 9,12-epithiostearate, 3,5,9-Trioxa-4-phosphatricosan-1-aminium, Eicosanoic acid,2-(1-oxohexadecyl)oxy, Hexa-t-butylselenatrisiletane, Glycocholic acid, 4-Piperidineacetic acid,1-acetyl-5-ethyl-2-[3-(2-hydroxyethyl)-1H-indol-2-yl]-à-methyl-, methylester, and Glycerol 2-acetate 1,3-dipalmitate. The data in the same table revealed that the addition of REO showed new compounds, which appeared in SLY samples and reflect the positive effect of REO addition. These compounds were found essentially in REO, including α-Pinene, Camphene, 3-Carene, Limonene, 1,6-Octadien-3-ol, 3,7-di methyl-, Camphor, Borneol, Bornyl acetate, Caryophyllene, Copaene, Retinol, Aromadendrene oxide-(2) and α-Guaiene, and the storage period declines these compounds. Furthermore, the data in the same table illustrate other compounds found in both REO and SLY control samples, such as octadecanal 2-bromo-, 1-Heptatriacotanol, psi.,.psi.-Carotene,1,1′,2,2′-tetrahydro-1,1′-dimethoxy, α-D-Glucopyranoside, methyl 2-(acetyl amino)-2-deoxy-3-O-(tri methyl silyl)-, cyclic methyl boronate, 2,4,6,8,10-Tetradecapentaenoic acid9a-(acetyloxy)-1a,1b,4,4a,5,7a,7b,8,9,9a-decahydro-4a,7b-dihydroxy-3-(hydroxymethyl)-1,1,6,8-tetramethyl-5-oxo-1H-cyclopropa(3,4)benz[1,2-e]azulen-9-ylester, Morphinan-4,5-epoxy-3,6-di-ol, 9,10-Secocholesta-5,7,10-triene-3,24,25-triol, 9-Hexadecenoic acid, Cyclopropanebutanoic acid,2-[[2-[[2-[(2-pentylcyclopropyl)methyl]cyclopropyl]methyl]cyclopropyl]methyl]-, methyl ester, 4aà,4bá-Gibbane-1à,10á-dicarboxylic acid, 3-Pyridinecarboxylic acid,2,7,10-tris(acetyloxy)-11a,2,3,4,6,7,10,11,11a-decahydro-1,1,3,6,9-penta methyl-4-oxo-4a,7aepoxy-5H-yclopenta[a]cyclopropa[f]cycloundecen-11-ylester,[1aR-], Agaricic acid, Oxiraneoctanoic acid,3-octyl-,cis-, Cinnamic acid, 4-hydroxy-3-methoxy-, Ocimene, Phenylalanine,4-amino-N-t-butyloxycarbonyl-, t-butylester, and 1-Monolinoleoylglycerol trim ethyl silyl ether, which all decreased with storage. In contrast, 9-Octadecenoic acid,(2-phenyl-1,3-dioxolan-4-yl) methyl ester trans-, Hexadecanoic acid,1-(hydroxyl methyl)-1,2-ethanediyl ester, 9,12,15-Octadecatrienoic acid, 2-phenyl-1,3-dioxan-5-yl ester, Butanoic acid,1a,2,5,5a,6,9,10,10a-octahydro, Dodecyl cis-9,10-epoxyoctadecanoate, 7,8-Epoxylanostan-11-ol, 3-acetoxy-, 4a-Phorbol 12,13-didecanoate, and 6,9,12,15-docosatetraenoic acid, methyl ester increased with storage. Meanwhile, other compounds were enhanced in SLY samples after REO addition, such as Docosanoic acid, 1,2,3-propanetriyl ester, Eucalyptol, and Glycine N-[(3à,5á,7à,12à)-24-oxo-3,7,12 tris[(trim ethyl silyl)oxy]cholan-24-yl]-,methyl ester (enhanced sample T2).

#### 3.3.2. Physico-Chemical Characterization

**Figure 2:**
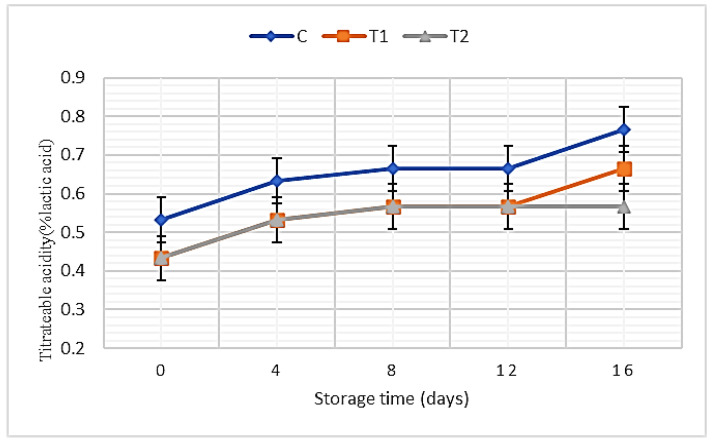
Acidity percentages of SLY samples without and with REO addition during storage up to 16 days.

- Acidity:

The data presented in Figure 2 illustrate the acidity percentages of SLY samples without and with REO addition. The obtained data showed significant differences between the control and SLY samples after REO addition. The T2 samples (with 0.7% REO addition) recorded the lowest value, while control samples recorded the highest value. Moreover, the acidity percentages increase with increasing the storage periods up to 16 days in all treatments, while samples containing essential oils were slower than control samples in the development of acidity.

**Figure 3:**
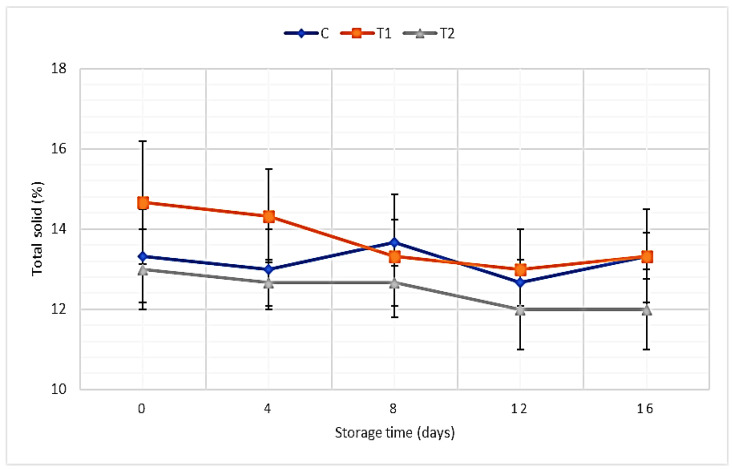
TS percentages of SLY samples without and with REO addition during storage up to 16 days.

- Total solids:

Data presented in Figure 3 illustrate the TS percentages of SLY samples without and with REO addition. The obtained data observed that TS percentages in SLY samples varied between 13 and 14.66%, and non-significant differences were found between control samples and other SLY samples after REO addition. Moreover, there were non-significant decreases in total solid content in all SLY samples during the storage period.

**Figure 4:**
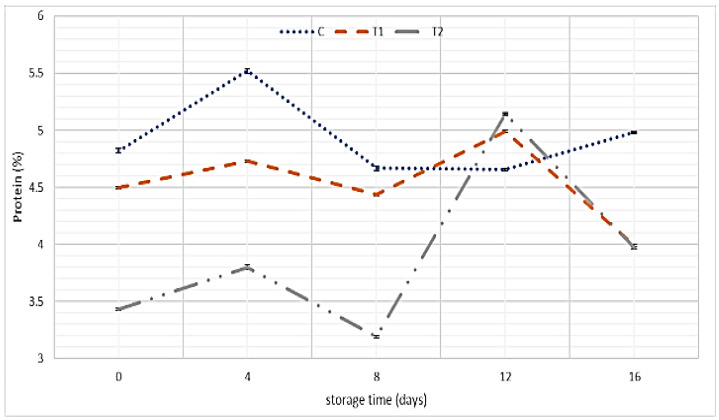
TP percentages of SLY samples without and with REO addition during storage up to 16 days.

- Total protein:

Data presented in Figure 4 illustrate the total protein (TP) percentages of SLY samples without and with REO addition. The obtained data showed that the addition of REO caused a significant decrease in TP percentages, while the control samples recorded the highest amount of TP percent up to 8 days of storage. However, the influence of REO addition on protein content appeared on day 12 of storage, as T1 and T2 samples recorded a higher value of protein content than control.

**Figure 5:**
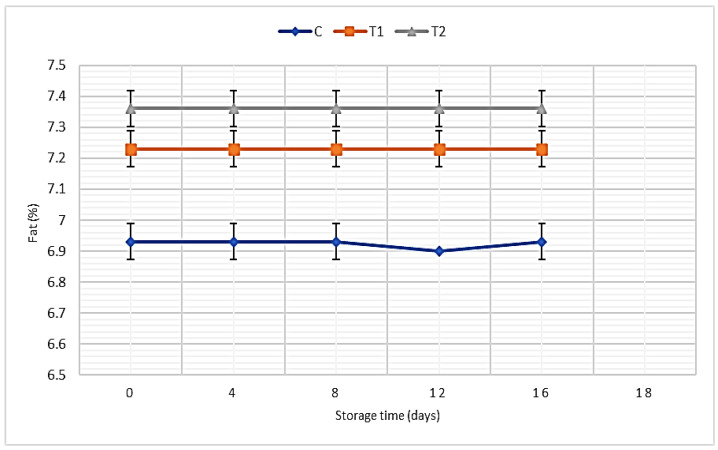
Fat percentages of SLY samples without and with REO addition during storage up to 16 days.

- Fat:

Data presented in Figure 5 illustrate the fat percentages of SLY samples without and with REO addition. The obtained data showed that the addition of REO caused a significant boost in fat content percentages, while the control samples recorded the lowest amount of fat percent, as the T2 samples recorded a much higher value. In contrast, data showed non-significant differences in the fat content during storage in all treatments.

#### 3.3.3. Microbiological Changes

Data presented in Table 4 illustrate the TPC as well as LAB (Log cfu/mL) of SLY samples without and with REO addition. The obtained data showed that the TBC and LAB increased significantly with increasing storage periods up to 16 days in all treatments. Moreover, control samples recorded the highest and lowest TBC and LAB, respectively. Furthermore, the addition of REO caused a substantial decrease and increase in TBC and LAB, respectively. The LAB count increased in all SLY samples during storage, indicating that REO addition boosted LAB count. Regarding yeasts and molds counts, the data showed that yeasts were detected in the control samples after 8 days of storage; they appeared after 12 days in SLY samples supplemented with REO (T1 and T2), while molds appeared only in the control sample after 8 days of storage, demonstrating the role of REO addition in slowing down the spoilage rate. On the other hand, the data observed that coliform group counts were not detected in all treatments. This might be due to the high-hygienic condition during the making of SLY and the development in the acidity.

#### 3.3.4. Sensory Evaluations

Data presented in Table 5 illustrate the organoleptic properties, such as flavor, body and texture, and appearance and overall acceptability of SLY samples, without and with REO addition. The obtained data showed that REO-supplemented samples had the greatest mean flavor, body and texture, and overall acceptance scores, while the control samples had the lowest scores. The data in the same table revealed that the impact of storage time on sensory characteristics, as the addition of REO (T1 and T2), improved flavor and general acceptability during storage up to 16 days in most treatments.

## 4. Discussion

### 4.1. Phytochemical Components in REO

#### 4.1.1. Total Phenolics and Total Flavonoids

Our data were lower than those of Adris et al., who found that total phenolic content was 29.23 mg gallic acid equivalent/g in the methanolic extract of rosemary [28]. Furthermore, Olmedo et al. found that REO contains 14.01 mg gallic acid/g of total phenol content [29]. The solvent used in oil extraction could explain the discrepancy. Adris et al. reported higher flavonoid content (6.59 mg catechin equivalent/g) in rosemary methanolic extract [28], when compared with our findings.

#### 4.1.2. The Chemical Analysis and Identification of REO by GC/MS

Olmedo et al. found that camphor (35.70 g/100 g) was the most important component in REO, followed by verbenone (26.20 g/100 g) and b-caryophyllene (15.80 g/100 g) (24). In addition, another study showed that rosemary oil contains 45 volatile compounds, and eucalyptol (1,8-cineole) was the most abundant (33.15%), followed by camphor (10.31%), α-pinene (8.11%), isocaryophyllene (7.02%), bornyl acetate (5.66%), α-terpineol (4.92%), and camphene (4.22%), while differences in geographic location [30] may explain the differences between our study and previous studies. Generally, the natural antioxidant properties are mainly attributed to their phenolic contents; thus, their antioxidants action is like synthetic phenolic antioxidants [31]. Moreover, phenolic compounds are well known as radical scavengers, metal chelators, reducing agents, hydrogen donors, and singlet oxygen quenchers [32]. Therefore, natural antioxidants can protect the human body from free radicals and could retard the progress of many chronic diseases, as well as lipid oxidative rancidity in foods [33]. In this respect, it might be that REO has an antioxidant effect, mainly attributed to its phenolic contents.

### 4.2. Antimicrobial Activity of REO

#### Minimum Inhibitory Concentration (MICs)

El-Kholy et al. studied the antimicrobial activity of REO against six microorganisms and found that the MIC of REO was 0.5%, with inhibition zones ranging from 7 to 13 mm in diameter [34]. A previous study by Jardak et al. found that REO has antibacterial activity against *Staphylococcus aureus* (1.25–2.50 µmL) [35]. In another investigation, by Fu et al., REO was found to have antimicrobial action against *Staphylococcus epidermidis*, *Escherichia coli,* and *Candida albicans*, with MICs ranging from 0.125 to 1.000% (*v*/*v*) [36]. In addition, Taheri et al. tested REO’s antibacterial effectiveness against six pathogens, finding that the REO effect on *Salmonella* sp., *Escherichia coli,* and *Staphylococcus aureus* was 9, 14, and 15 mm for the zone of inhibition, respectively [37]. REO demonstrated activity against *E. coli* and *Staph. aureus* with 1.25 and 5 µmL, respectively [38]. Another study, by Bousbia et al., found that REO has antibacterial action against *Staphylococcus aureus* and *Escherichia coli*, with inhibition zones 12.5 and 15.5 mm, respectively [39]. The presence of 1,8-cineole (eucalyptus) and α-terpineol exhibited a significant antibacterial effect [40]. On the other hand, the inhibition zone for REO against *E. coli* was 16.3 ± 0.6 mm [41]. The antimicrobial activity of REO against *Staphylococcus aureus* was 10.33 mm, and against *Candida albicans* was 8 mm inhibitory zone diameter [42], which was lower than our findings, due to changes in essential oil extraction procedures [43]. The stronger antifungal activity against *Candida albicans* was when utilizing 160 μL-1 of REO resulted in inhibitory zones of nearly 68 mm [44]. The existence of α-pinene content in REO caused antifungal activity [45], Also Kabouche et al. [46] referred to the antibacterial and antifungal effect of REO extract. Generally, the inhibition zone increased with increasing REO concentration in all studied bacteria, especially in *Staphylococcus aureus*. Thus, they achieved the highest inhibition zones (14 mm) at 100% of REO. However, low concentrations of REO weakly inhibited the development of tested strains. Comparatively, the *Escherichia coli* strain was low in sensitivity to the inhibitory activity of the REO compared to that of all studied bacteria in all concentrations. These results are in agreement with those reported by Zakia, who proposed that Gram-positive bacteria are more resistant than Gram-negative bacteria to the antibacterial properties of plant volatile oils [47]. Furthermore, the work of Deans et al. is in contrast to the hypothesis proposed that the susceptibility of bacteria to plant volatile oils and the Gram reaction appears to have little influence on growth inhibition [48].

### 4.3. The effect of REO Addition on the Shelf-Life of SLY

#### 4.3.1. Gas Chromatography-Mass Spectrometry (GC-MS) Analysis

Obviously, many compounds in the plain SLY control sample (C) decreased with storage, such as Octadecane, 3-ethyl-5-(2-ethylbutyl), which has antioxidant and anti-inflammatory effect [49], 1-Nonadecene, which has antifungal and anticancer effects [50], Phthalic acid derivatives, which have a role in chronic cardiovascular and cerebrovascular diseases treatments and illustrated its ability in antitumor, anti-inflammatory, and antibacterial activities [51] and Ethanol, 2-(octadecyloxy) (which has antimicrobial effect [52])The data in our study revealed that the addition of REO showed new compounds, such as Camphene, which inhibits the biosynthesis of cholesterol and has a promising potential as a lipid-lowering agent [53], 3-Carene which shows a protective effect in postharvest pathogens [54], Limonene, which has lemongrass herbal aroma, and has a curative effect on heartburn and gastroesophageal reflux [55,56], 1,6-Octadien-3-ol, 3,7-di methyl-, which has anti-inflammatory and anti-cancer properties [49], Camphor, which has an important role in cough and colds cure [57], Bornyl acetate, which has pine, woody, and camphoreous flavors [58,59], Caryophyllene, which has antitumor, analgesic, antibacterial, anti-inflammatory, sedative, and fungicide properties [60], Copaene (causes an increase in antioxidant capacity of human lymphocyte [61]), Retinol (which is converted in the human body and is important in vision physiology [62]), and Aromadendrene oxide-(2) (which has anticancer activity [63]); however, the storage period declines these compounds. Furthermore, our data illustrate other compounds found in both REO and SLY control samples, which decreased with storage, such as Octadecanal 2-bromo- (which has anti-inflammatory and anti-apoptotic effects [64]), 1-Heptatriacotanol (which has antioxidant, anticancer and anti-inflammatory effects [65]), Cyclic methyl boronate (which has a preservative activity [60]), 3-Pyridinecarboxylic acid,2,7,10-tris(acetyloxy)-11a,2,3,4,6,7,10,11,11a-decahydro-1,1,3,6,9-penta methyl-4-oxo-4a,7aepoxy-5H-yclopenta[a]cyclopropa[f]cycloundecen-11-ylester,[1aR-], which has anti-inflammatory effect [64], Cinnamic acid, 4-hydroxy-3-methoxy- (which has a promising potential as an anticancer treatment) [66], Ocimene (has a sweet herbal odor [67]), and 1-Monolinoleoylglycerol trim ethyl silyl ether (which has antimicrobial activity [68]); in contrast, other compounds increase with storage, such as 9-Octadecenoic acid,(2-phenyl-1,3-dioxolan-4-yl) methyl ester trans (which has antimicrobial and anti-inflammatory properties [69]), 9,12,15-Octadecatrienoic acid, 2-phenyl-1,3-dioxan-5-yl ester (which has antimicrobial and anti-inflammatory properties [69]), Butanoic acid,1a,2,5,5a,6,9,10,10a-octahydro (indeed, acid compounds (including Butanoic acid) increased after either 3 or 7 days of storage [70]), 7,8-Epoxylanostan-11-ol, 3-acetoxy- (which has antimicrobial and anti-inflammatory effects [71,72]), and 6,9,12,15-docosatetraenoic acid, methyl ester (which has anti-carcinogenic and anti-atherosclerotic effects [73]). Meanwhile, other compounds were enhanced in SLY samples after REO addition, such as ester compounds (even at low concentrations, it contributed to the flavors of dairy products [74]), Eucalyptol, which has a eucalyptus, bark lavender, fresh mint -like fragrance, spicy aroma, and taste [52,55,56,57,58,59,60,61,62,63,64,65,66,67,68,69,70,71,72,73,74,75], and Glycine N-[(3à,5á,7à,12à)-24-oxo-3,7,12 tris[(trim ethyl silyl)oxy]cholan-24-yl]-,methyl ester (which has antibacterial and antiperspirant activities [76]).

#### 4.3.2. Physico-Chemical Characterization

- Acidity:

Ali et al. found that the addition of rosemary extracts during yogurt manufacturing affected the Gram-positive bacterial cell surface and inhibited its growth [77]. Likewise, the addition of oregano and rosemary essential oils to cream cheese declines the acidity value when compared with control samples [29]. Moreover, yogurt fortified with fish oil/γ-oryzanol showed a lower acidity than the plain yogurt [78]. The acidity value increased significantly during storage, and similar findings were noticed by El-Kholy et al. [34]. Indeed, the acidity percentages increased during storage due to the increase in lactic acid bacteria counts, which converted the lactose into lactic acid [79], whereas previous study found that the addition of REO to the yogurt samples did not show differences in titratable acidity and pH values during the storage period, and the titratable acidity of the control samples increased substantially [15].

- Total solid:

Our results are in agreement with Al-Soudy et al. [80], who found non-significant differences between the control sample and drinking yoghurt samples (infused with herbal extract) in total solids, due to the little quota of the herbal extract, whereas El-Sayed et al. found that fortified Labneh with *Moringa oleifera* oil increased the TS percentages [81]. Our results showed a non-significant decrease in total solid content during the storage period; similar results were obtained by Ghalem and Zouaoui, who found that dry matter percentage decreased in control yogurt samples with storage period [15].

- Total protein:

Results obtained by El-Kholy et al., who found that the protein content decreased in cheese samples after the addition of REO (from 17.18% in control samples to 16.91% in cheese made with REO) [34], were in agreement with our study. The decline in protein content in our study after 8 days of storage could be attributed to proteolytic activity by added microorganisms (76). Our findings are consistent with the results of El-Kholy et al. and Thabet et al. [34,82].

- Fat:

Our results were consistent with a previous study, conducted by Ghalem and Zouaoui, as they observed a slight increase in yogurt fat with REO addition [15]. Moreover, the addition of REO extracts to the cheese showed a significant (*p* ≤ 0.05) higher fat content than the control cheese samples [34]. Our data illustrated that fat content during storage matched the results reported by Ghalem and Zouaoui [15].

#### 4.3.3. Microbiological Changes

Generally, the data revealed that there were decreases in TBC and yeasts and molds counts in SLY with increasing REO levels in all treatments. This may be due to the effect of REO on TBC and yeasts and molds. Indeed, REO contains monoterpenes, such as α-pinene, 1,8-cineole, and borneol (Table 1), which have strong antibacterial and antimicrobial activities on food product deterioration pathogens [83].

Control samples had higher counts of TBC and yeasts and molds than those of SLY with REO in all treatments. Similar findings were reported by El-Kholy et al. [34]. On the other hand, Ali et al. [77] discovered that rosemary extract had a detrimental effect on the starter cultures *Streptococcus thermophilus* and *Lactobacillus delbrueckii* subsp. *bulgaricus*. Our data illustrated the effect of REO addition in slowing down the spoilage rate and similar observations were reported by Ghalem and Zouaoui and Ali et al. [15,77].

#### 4.3.4. Sensory Evaluations

The enhancement effect of REO addition on sensory properties in our study matched the findings reported by El-Kholy et al., as they showed that adding REO to ultra-filtrated soft cheese samples improved sensory characteristics, while the control samples had lower scores [34]. According to other findings, adding 0.14 g/L of REO to yogurt samples resulted in the best flavor, taste, and texture, whereas increasing the previous concentration resulted in the worst flavor, taste, and texture [15]. The control samples degraded in all sensory quality properties due to aromatic chemicals (such as acetone and acetaldehyde) and lactic acid generation during storage [84]. These results are in agreement with those reported by Ali et al., who proposed that the addition of aqueous extract of rosemary affected the sensory properties of yogurt (flavor, body and texture, appearance, and overall grade), wherein an increasing concentration of rosemary extract increased the score of flavor, body and texture, appearance, and overall grade [77].

## 5. Conclusions

The results of antibacterial characteristics of REO indicated that it could be used as a natural preservative in the manufacture of SLY. In addition, SLY samples with REO addition showed acceptable flavor, body and texture, appearance, and overall acceptance. Based on the analysis, SLY with REO at 0.5% and 0.7% performed equally in this study, which indicates that adding REO at 0.7% would not provide additional benefits when compared with REO at 0.5% in SLY. Therefore, REO at 0.5% could be utilized in the manufacture of SLY.

## Figures and Tables

**Table 1:** Gas chromatography-Mass Spectrometry (GC-MS) analysis results of REO.

Peak No.	RT* (min)	Compound Name	Area%
1	5.81	Octadecanal, 2-bromo-	0.0126
2	7.32	9-Hexadecenoic acid	0.0295
3	7.6	Morphinan-4,5-epoxy-3,6-di-ol 6-[7-nitrobenzofurazan-4-yl]amino-	0.0077
4	8.79	2,4,6,8,10-Tetradecapentaenoic acid9a-(acetyloxy)-1a,1b,4,4a,5,7a,7b,8,9,9a-decahydro-4a,7b-dihydroxy-3-(hydroxyl methyl)-1,1,6,8-tetra methyl-5-oxo-1H-cyclopropa[3,4]benz = [1,2-e]azulen-9-ylester,	0.0061
5	9.82	Ocimene	0.0209
6	10.9	Tricyclo[2.2.1.0(2,6)]heptane,1,7,7-tri methyl-	0.2565
7	11.46	α-Pinene	15.3175
8	11.55	3-Carene	8.2993
9	12.56	Camphene	8.3843
10	16.13	α-Phellandrene	0.3851
11	16.56	1R-à-Pinene	0.2715
12	17.56	Limonene	0.0053
13	17.8	Eucalyptol	9.3686
14	18.11	Trifluoroacetyl-α-terpineol	3.6107
15	19.14	Phenylalanine,4-amino-N-t-butyloxycarbonyl-, t-butylester	0.0015
16	19.47	2-Furanmethanol,	0.0232
17	19.88	à-D-Glucopyranoside, methyl	0.0034
18	20.19	Bicyclo[4.1.0]hept-2-ene,	0.7319
19	20.34	1,6-Octadien-3-ol, 3,7-di methyl-	2.1247
20	20.54	trans-Z-α-Bisabolene epoxide	0.0061
21	20.88	Oxiraneoctanoic acid, 3-octyl-, cis-	0.0049
22	21.17	2,5-Octadecadiynoic acid, methyl ester	0.0105
23	21.42	Fenchol, exo-	0.1846
24	21.85	Camphenol, 6-	0.0987
25	22.36	2,4,6-Decatrienoic acid, 1a,2,5,5a,6,9,10,10a-octahydro-5,5a-di hydroxy-4-(hydroxyl methyl)-1,1,7,9-tet ramethyl-11-oxo-1H-2,8a-methanocycl openta[a]cyclopropa[e]cyclodecen-6-yl ester, [1aR-(1aà,2à,5á,5aá,6á,8aà,9à,10aà)]-	0.0057
26	22.7	Isopinocarveol	0.0103
27	23.17	Bicyclo[2.2.1]heptan-2-one, 1,7,7-tri methyl-, (1R)- (camphor)	21.8229
28	24.03	Borneol	8.1423
29	24.36	3-Cyclohexen-1-ol,	1.2918
30	24.97	3-Cyclohexene-1-methanol, à,à4-tri methyl-	4.4136
31	25.34	(-)-Myrtenol	0.0170
32	25.53	9-Octadecenoic acid,(2-phenyl-1,3-dioxolan-4-yl)methyl ester, trans-	0.0117
33	26.17	6-Octen-1-ol, 3,7-di methyl-	0.0756
34	26.68	Ingol 12-acetate	0.0103
35	27.23	Isobornyl formate	0.0161
36	27.77	Geranyl vinyl ether	0.0391
37	28.7	E,E,Z-1,3,12-Nonadecatriene-5,14-diol	0.0096
38	29.04	Linoleic acid ethyl ester	0.0120
39	29.54	2,2,4-Tri methyl-3-(3,8,12,16-tetrameth yl-heptadeca-3,7,11,15-tetraenyl)-cycl ohexanol	0.0072
40	29.75	Thymol	0.0303
41	30.65	Bornyl acetate	1.3593
42	31	Phenol, 2-methyl-5-(1-methyl ethyl)-	0.0488
43	31.77	9,10-Secocholesta-5,7,10(19)-triene-3,	0.0013
44	32.6	2-Butenoic acid, 2-methyl-,2-(acetyloxy)-1,1a,2,3,4,6,7,10,11,11adecahydro-7,10-dihydroxy-1,1,3,6,9-pe ntamethyl-4a,7a-epoxy-5H-cyclopenta[a]cyclopropa[f]cycloundecen-11-yl ester, [1aR-[1aR*,2R*,3S*,4aR*,6S*,7S*,7aS	0.0127
45	33.05	Doconexent	0.0091
46	34.15	Gibberellic acid	0.0063
47	34.61	Eugenol	0.2061
48	35.49	Retinol	0.0006
49	35.9	Ylangene	0.1776
50	36.13	Copaene	0.8834
51	36.69	1H-Cycloprop[e]azulene, decahydro-1,1,7-tri methyl-4-methylene-, [1aR-(1aà,4aá,7à,7aá,7bà)]	0.0679
52	37.31	Androstan-17-one, 3-ethyl-3-hydroxy-, (5à)-	0.0120
53	38.46	Caryophyllene	9.8533
54	38.7	Aromadendrene	0.0999
55	39.52	Humulen-(v1)	0.1179
56	39.83	α-Caryophyllene	1.1039
57	40.87	1,6-Cyclodecadiene,1-methyl-5-methylene-8-(1-methylethyl)-, [s-(E,E)]-	0.0494
58	41.32	Longifolene-(V4)	0.2488
59	41.73	Seychellene	0.0396
60	42.46	1H-Indene, 2,3-dihydro-1,1,5,6-tetra methyl-,	0.0118
61	42.84	α-Cubebene	0.0368
62	43.06	α-Guaiene	0.0280
63	43.42	α-Calacorene	0.0713
64	44.17	6,9,12,15-Docosatetraenoic acid, methyl ester	0.0395
65	45.14	Cyclopropanebutanoic acid,	0.0018
66	45.79	Caryophyllene oxide	0.0771
67	46.5	Pseudosolasodine diacetate	0.0061
68	47.13	Cubenol	0.0321
69	47.69	Patchoulene	0.0168
70	47.91	Methyl jasmonate	0.0093
71	48.35	.tau.-Cadinol	0.0818
72	49.5	Longipinocarveol, trans	0.0409
73	50.33	1H-2,8a-Methanocyclopenta[a]cyclopr opa[e]cyclodecen-11-one, 1a,2,5,5a,6,9,10,10a-octahydro-5,5a,6-t rihydroxy-1,4-bis(hydroxyl methyl)-1,7,9-tri methyl-, [1S-(1à,1aà,2à,5á,5aá,6á,8aà,9à,10aà)]-	0.0341
74	54.21	Cinnamic acid, 4-hydroxy-3-methoxy-,	0.0007
75	55.01	Agaricic acid	0.0008
76	55.6	7aH-Cyclopenta[a]cyclopropa[f]cycloundecene-2,4,7,7a,10,11-hexol,1,1a,2,3,4,4a,5,6,7,10,11,11a-dodecahydro-1,1,3,6,9-penta methyl-,2,4,7,10,11-pentaacetate	0.0160
77	59.2	Dodecyl cis-9,10-epoxyoctadecanoate	0.0038
78	59.7	Butanoic acid,1a,2,5,5a,6,9,10,10a-octahydro	0.0022
79	60.97	1-Heptatriacotanol	0.0143
80	61.56	Prednisone	0.0114
81	62.69	Docosanoic acid, 1,2,3-propanetriyl ester	0.0095
82	64.12	2-(16-Acetoxy-11-hydroxy-4,8,10,14-tetra methyl-3-	0.0031
83	64.63	4aà,4bá-Gibbane-1à,10á-dicarboxylic acid	0.0007
84	65.03	7,8-Epoxylanostan-11-ol, 3-acetoxy	0.0018
85	65.42	4a-Phorbol 12,13-didecanoate	0.0020
86	70.37	Hexadecanoic acid,1-(hydroxyl methyl)-1,2-ethanediyl ester	0.0059
87	71.68	1-Monolinoleoylglycerol tri methyl silyl ether	0.0142
88	74.31	psi.,.psi.-Carotene,1,1′,2,2′-tetrahydro-1,1′-dimethoxy-	0.0008
89	74.73	Glycine N-[(3à,5á,7à,12à)-24-oxo-3,7,12-tris[(tri methyl silyl)oxy]cholan-24-yl]-,methyl ester	0.0062
90	75.44	9,12,15-Octadecatrienoic acid,	0.0007
91	75.66	3-Pyridinecarboxylic acid,2,7,10-tris(acetyloxy)-1,1a,2,3,4,6,7,10,11,11a-decahydro-1,1,3,6,9-penta methyl-4-oxo-4a,7a-epoxy-5H-cyclopenta[a]cyclopropa[f]cycloundecen-11-ylester,[1aR-]	0.0059

RT*: Retention time.

**Table 2:** Minimum inhibitory concentration (MICs) of REO with some species of microorganisms.

	REO Concentration (%)	12.5	25	50	100	DMSO *
Microorganisms		Inhibition Zone (mm)				
***Escherichia coli* (G-ve) AUMC No. B-53**		0	6	7	13	0
***Salmonella marcescens* (G-ve) AUMC No. B-**		0	6	7	13	0
***Staphylococcus aureus* (G + ve) AUMC No. B-54**		0	6	8	14	0
***Aspergillus flavus* AUMC No. 1276**		Nd	Nd	Nd	7	0
***Candida albicans* AUMC No. 9160**		Nd	Nd	Nd	9	0

The amount added in each pore is 50 µL; Nd: Not determined; * DMSO: Negative control by dimethyl sulfoxide.

**Table 3:** Gas chromatography (GC)-Mass Spectrometry (MS) analysis results of SLY samples without and with REO addition.

No	Compounds/Treatments	C		T1		T2	
		Fresh	After 16 Days	Fresh	After 16 Days	Fresh	After 16 Days
1	Octadecanal, 2-bromo-	2.807	0	0.593	0.566	0.292	0.292
2	9-Octadecenoic acid,(2-phenyl-1,3-dioxolan-4-yl) methyl ester, trans-	0.265	0.455	0.197	0.299	0.126	0.118
3	1-Heptatriacotanol	1.347	0.381	0.264	0.448	0.182	0.079
4	psi.,.psi.-Carotene,3,4-didehydro-1,2-dihydro-1-methoxy	0	0.692	0	0.508	0.419	0.161
5	psi.,.psi.-Carotene,1,1′,2,2′-tetrahydro-1,1′-dimethoxy-	3.226	0	1.382	0	0.183	0
6	Docosanoic acid, 1,2,3-propanetriyl ester	0.906	0.830	2.175	5.428	2.583	7.619
8	Eucalyptol	4.681	2.632	45.936	37.258	48.772	40.417
9	α-Pinene	0	0	8.308	4.609	7.069	3.610
11	α-D-Glucopyranoside, methyl2-(acetyl amino)-2-deoxy-3-O-(tri methyl silyl)-, cyclic methyl boronate	0.366	0	0.057	0.068	0.065	0.114
12	2,4,6,8,10-Tetradecapentaenoic acid9a-(acetyloxy)-1a,1b,4,4a,5,7a,7b,8,9,9a-decahydro-4a,7b- dihydroxy-3-(hydroxymethyl)-1,1,6,8-tetramethyl-5-oxo-1H-cy clopropa[3,4]benz[1,2-e]azulen-9-ylester	0.605	0.149	0.121	0.142	0.068	0.050
13	Morphinan-4,5-epoxy-3,6-di-ol6-[7-nitrobenzofurazan-4-yl]amino-	0.408	0.238	0	0.040	0.046	0
14	Oleic acid, eicosyl ester	2.366	2.949	0.414	2.188	0.588	1.065
15	Geranyl isovalerate	0.828	0.409	0.239	0	0	0
16	9,10-Secocholesta-5,7,10-triene-3,24,25-triol,	0.961	0.124	0.726	0	0.802	1.967
17	Bis(benzimidazol-2-ylmethyl)sulfone	0	0.218	0.180	0.166	0	0
18	9-Hexadecenoic acid	1.017	0.895	0.252	0.314	0	0
19	Hexadecanoic acid,1-(hydroxyl methyl)-1,2-ethanediyl ester	1.318	1.525	0.675	1.259	0.601	0.541
20	Cyclopropanebutanoic acid,2-[[2-[[2-[(2-pentylcyclopropyl)methyl]cyclopropyl]methyl]cyclopropyl]methyl]-, methyl ester	2.429	0.778	0.447	0.558	0.313	0.275
21	4aà,4bá-Gibbane-1à,10á-dicarboxylic acid,	1.438	0.327	0.274	0.511	0.182	0.088
22	3-Pyridinecarboxylic acid,2,7,10-tris(acetyloxy)-1,1a,2,3,4,6,7,10,11,11a-decahydro-1,1,3,6,9-penta methyl-4-oxo-4a,7a-epoxy-5H-cyclopenta[a]cyclopropa[f]cycloundecen-11-ylester,[1aR-]	2.093	0.284	0.300	0.169	0.208	0.047
23	Octadecane, 3-ethyl-5-(2-ethylbutyl)-	2.223	0.542	0.356	0.342	0.221	0.191
24	Agaricic acid	0.876	0.186	0.146	0.131	0.093	0.175
25	Phenol, 2,4-bis(1,1-di methyl ethyl)	42.117	11.887	7.059	7.005	4.473	4.186
26	1-Nonadecene	10.516	2.005	1.867	2.188	1.256	1.154
27	7-Methyl-Z-tetradecen-1-ol acetate	1.020	0.294	0.178	0.188	0.114	0.160
28	9,12,15-Octadecatrienoic acid, 2-phenyl-1,3-dioxan-5-yl ester	2.916	1.173	0.599	3.534	0.456	0.461
29	Oxiraneoctanoic acid, 3-octyl-, cis-	0.838	0.215	0.202	0.223	0.125	0.097
30	Cinnamic acid, 4-hydroxy-3-methoxy-,	0.477	0.202	0.155	0	0.111	0.068
31	Cholest-22-ene-21-ol,	0	0.249	0.091	0.168	0.159	0.129
32	Phorbol	0.117	0.235	0	0.124	0.0548	0
33	Bacteriochlorophyll-c-stearyl	0	3.328	0	2.016	1.386	0.678
34	Butanoic acid,1a,2,5,5a,6,9,10,10a-octahydro	0	0.136	0	0	0	0.0924
35	Phthalic acid, butyl undecyl ester	2.629	0.841	0.487	0.787	0.339	0.322
36	Dodecyl cis-9,10-epoxyoctadecanoate	0	5.232	0	2.259	0	5.654
37	Pregn-5-ene-3,11-dione,17,20:20,21-	0.318	0.588	0.122	0.063	0.125	0.207
38	Methyl 9,12-epithiostearate	0	0.830	0	0.477	0	0
39	3,5,9-Trioxa-4-phosphatricosan-1-aminium	0	0.542	0	1.791	0	0.183
40	Eicosanoic acid,2-[(1-oxohexadecyl)oxy]-	0	21.207	0	0.814	0	2.801
41	7,8-Epoxylanostan-11-ol, 3-acetoxy	0.548	7.066	0.105	0.562	0.053	1.224
42	Hexa-t-butylselenatrisiletane	0	4.938	1.086	0	1.110	2.424
43	4a-Phorbol 12,13-didecanoate	0.161	0.224	0.059	1.511	0.128	0.111
44	Glycocholic acid	0	1.068	0	3.151	0	0
45	Acetic acid,17-(4-hydroxy-5-methoxy-1,5-di methyl hexyl)-4,4,10,13,14-penta methyl	0.268	12.329	0.081	0.0451	0.0534	0.568
46	4-Piperidineacetic acid,1-acetyl-5-ethyl-2-[3-(2-	0	2.215	0	1.022	0	0.866
47	Glycerol 2-acetate 1,3-dipalmitate	0	9.230	0	0.206	0	2.633
48	Camphene	0	0	2.463	1.452	1.957	1.082
49	Ocimene	0.937	0	0.287	0.307	0.226	0.219
50	3-Carene	0	0	0.130	0	0.098	0
51	Limonene	0	0	1.459	0.866	1.225	0.606
52	Octadecanoic acid,(2-phenyl-1,3-dioxolan-4-yl)methyl ester, cis-	1.017	0.145	0.185	0.097	0.125	0.051
53	1,6-Octadien-3-ol, 3,7-dimethyl-	0	0	0.942	0.680	0.941	0.693
54	Camphor	0	0	10.046	8.242	11.340	10.249
55	6,9,12,15-Docosatetraenoic acid, methyl ester	0.842	0	0.281	0.352	0	0.276
56	Borneol	0	0	3.748	2.850	4.018	3.397
57	Phenylalanine,4-amino-N-t-butyloxycarbonyl-, t-butylester	0.531	0	0.072	0	0	0.105
58	Tetradecanoic acid,3,3a,4,6a,7,8,9,10,10a	0.542	0	0.070	0.131	0.055	0.068
59	Bornyl acetate	0	0	0.508	0	0.541	0.375
60	Caryophyllene	0	0	2.757	1.654	2.564	1.757
61	Copaene	0	0	0.230	0	0.218	0.136
62	Retinol	0	0	0.103	0	0.102	0.154
63	Aromadendrene oxide-(2)	0	0	0.462	0	0.417	0
64	α-guaiene	0	0	0.200	0	0.195	0
65	Estra-1,3,5(10)-trien-17á-ol	0.733	0	0.049	0.151	0.045	0
66	Ethanol, 2-(octadecyloxy)-	1.437	0	0.356	0	0	0
67	2-(16-Acetoxy-11-hydroxy-4,8,10,14-tetra methyl-3-	0.443	0	0.068	0.071	0.063	0
68	Glycine N-[(3à,5á,7à,12à)-24-oxo-3,7,12-tris[(tri methyl silyl)oxy]cholan-24-yl]-,methyl ester	0.332	0	0.153	0	2.583	0
69	1-Monolinoleoylglycerol trim ethyl silyl	1.425	0	0.290	0	0.525	0

**Table 4:** Effect of REO on microbiological properties (Log cfu/mL) of SLY during storage periods (mean ± standard deviation).

Microbial Type	Storage Time (d)	Treatments		
		Control	T1	T2
**Total bacterial** **count**	**Fresh**	7.24 ± 0.24	7.24 ± 0.010	7.25 ± 0.010
	**4**	7.47 ± 0.373	7.34 ± 0.010	7.30 ± 0.010
	**8**	8.09 ± 0.090	7.86 ± 0.117	7.80 ± 0.179
	**12**	8.51 ± 0.036	8.42 ± 0.040	8.31 ± 0.036
	**16**	8.66 ± 0.056	8.55 ± 0.055	8.506 ± 0.112
	**Mean**	7.99 ^a^	7.88 ^b^	7.83 ^b^
**Lactic acid bacteria**	**Fresh**	7.02 ± 0.02	7.10 ± 0.01	7.20 ± 0.01
	**4**	7.40 ± 0.01	7.50 ± 0.02	7.70 ± 0.02
	**8**	8.18 ± 0.02	8.55 ± 0.20	8.64 ± 0.13
	**12**	8.37 ± 0.01	8.73 ± 0.06	8.82 ± 0.05
	**16**	8.39 ± 0.01	8.75 ± 0.07	8.89 ± 0.01
	**Mean**	7.87 ^c^	8.13 ^b^	8.25 ^a^
**Yeasts count**	**Fresh**	0.00	0.00	0.00
	**4**	0.00	0.00	0.00
	**8**	6.88 ± 0.04	0.00	0.00
	**12**	7.62 ± 0.24	7.10 ± 0.10	6.77 ± 0.06
	**16**	8.04 ± 0.35	7.20 ± 0.10	6.84 ± 0.01
	**Mean**	4.506 ^a^	2.86 ^b^	2.72 ^c^
**Molds count**	**Fresh**	ND	ND	ND
	**4**	ND	ND	ND
	**8**	6.20 ± 0.17	ND	ND
	**12**	6.26 ± 0.24	ND	ND
	**16**	6.26 ± 0.24	ND	ND
	**Mean**	3.74 ^a^	0.00 ^b^	0.00 ^b^
**Coliform count**	**Fresh**	ND*	ND	ND
	**4**	ND	ND	ND
	**8**	ND	ND	ND
	**12**	ND	ND	ND
	**16**	ND	ND	ND
	**Mean**	ND	ND	ND

ND*: Not Detected; ^a–c^ Means in the same row not sharing a common superscript are statistically different at *p* < 0.05.

**Table 5:** Sensory quality properties of SLY samples without and with REO addition (mean ± standard deviation) during storage periods up to 14 days.

Items	Storage Time (d)	Treatments			SEM
		Control	T1	T2	
**Flavor**	**Fresh**	39.00 ± 1.00	40.00 ± 1.00	40.33 ± 0.577	0.32
	**4**	38.00 ± 1.00	41.33 ± 0.577	41.33 ± 0.577	0.60
	**8**	36.33 ± 0.577	42.00 ± 1.00	42.00 ± 1.00	0.98
	**12**	35.00 ± 1.00	43.00 ± 1.00	43.00 ± 1.00	1.36
	**16**	33.00 ± 1.00	43.67 ± 0.577	43.33 ± 0.577	1.76
	**Mean**	36.27 ^b^	42.00 ^a^	42.00 ^a^	0.48
**Body and** **texture**	**Fresh**	12.00 ± 0.577	12.33 ± 1.00	13.00 ± 0.577	0.29
	**4**	11.00 ± 0.577	12.33 ± 1.00	12.33 ± 0.577	0.42
	**8**	10.33 ± 0.577	12.00 ± 0.577	12.67 ± 0.577	0.47
	**12**	9.33 ± 1.00	13.00 ± 1.00	13.00 ± 1.00	0.66
	**16**	9.00 ± 1.00	13.33 ± 1.00	12.67 ± 1.155	0.75
	**Mean**	10.33 ^b^	12.60 ^a^	12.73 ^a^	0.24
**Appearance**	**Fresh**	12.00 ± 1.00	12.33 ± 0.577	13.00 ± 1.00	0.29
	**4**	11.00 ± 1.00	12.33 ± 1.155	12.33 ± 1.528	0.42
	**8**	10.33 ± 0.577	12.00 ± 1.00	12.67 ± 1.528	0.47
	**12**	9.33 ± 0.577	13.00 ± 1.00	13.00 ± 1.00	0.66
	**16**	9.00 ± 1.00	13.33 ± 0.577	12.67 ± 1.528	0.75
	**Mean**	10.33 ^b^	12.60 ^a^	12.73 ^a^	0.24
**Overall acceptability**	**Fresh**	87.33 ± 1.155	89.33 ± 2.082	91.00 ± 1.00	0.68
	**4**	84.67 ± 1.155	89.67 ± 1.155	90.33 ± 0.577	0.94
	**8**	81.33 ± 0.577	89.67 ± 1.528	91.00 ± 3.00	1.62
	**12**	78.33 ± 1.528	92.00 ± 1.00	93.00 ± 2.00	2.41
	**16**	75.00 ± 1.00	94.00 ± 1.00	92.67 ± 2.082	3.09
	**Mean**	81.33 ^b^	90.93 ^a^	91.60 ^a^	0.84

^a–c^ means in the same row not sharing a common superscript are different at *p* < 0.05. SEM: standard error of the mean.

## Data Availability

Data are contained within the article.

## References

[1] Inanli A.G., Tümerkan E.T.A., El-Abed N., Regenstein J.M., Özogul F. (2020). The impact of chitosan on seafood quality and human health: A review. Trends Food Sci. Technol..

[2] Stratford M., Amparo Q., Graham H.F. (2006). Food and beverage spoilage yeasts. Yeasts in Food and Beverages.

[3] Burt S.A., Fledderman M.J., Haagsman H.P., van Knapen F., Veldhuizen E.J.A. (2007). Inhibition of Salmonella enterica serotype Enteritidis on agar and raw chicken by carvacrol vapour. Int. J. Food Microbiol..

[4] Tager L.R., Krause K.M. (2011). Effects of essential oils on rumen fermentation, milk production, and feeding behavior in lactating dairy cows. J. Dairy Sci..

[5] Berardini N., Knödler M., Schieber A., Carle R. (2005). Utilization of mango peels as a source of pectin and polyphenolics. Innov. Food Sci. Emerg. Technol..

[6] Belletti N., Kamdem S.S., Tabanelli G., Lanciotti R., Gardini F. (2010). Modeling of combined effects of citral, linalool and β-pinene used against Saccharomyces cerevisiae in citrus-based beverages subjected to a mild heat treatment. Int. J. Food Microbiol..

[7] Mohammed M.J., Anand U., Altemimi A.B., Tripathi V., Guo Y., Pratap-Singh A. (2021). Phenolic Composition, Antioxidant Capacity and Antibacterial Activity of White Wormwood (Artemisia herba-alba). Plants.

[8] Pinela J., Prieto M.A., Barreiro M.F., Carvalho A.M., Oliveira M.B.P., Vázquez J.A., Ferreira I.C.F.R. (2016). Optimization of microwave-assisted extraction of hydrophilic and lipophilic antioxidants from a surplus tomato crop by response surface methodology. Food Bioprod. Process..

[9] Lo Presti M., Ragusa S., Trozzi A., Dugo P., Visinoni F., Fazio A., Dugo G., Mondello L. (2005). A comparison between different techniques for the isolation of rosemary essential oil. J. Sep. Sci..

[10] Siejak P., Smułek W., Fathordobady F., Grygier A., Baranowska H.M., Rudzinska M., Masewicz Ł., Jarzebska M., Nowakowski P.T., Makiej A. (2021). Multidisciplinary Studies of Folk Medicine “Five Thieves’ Oil” (Olejek Pieciu Złodziei) Components. Molecules.

[11] Bourlioux P., Pochart P. (1988). Nutritional and Health Properties of Yogurt. World Rev. Nutr. Diet.

[12] Sahana N., Yasarb K., Hayaloglu A.A. (2008). Physical, chemical and flavour quality of non-fat yogurt as affected by ab-glucan hydrocolloidal composite during storage. Food Hydrocoll..

[13] Penna A.L.B., Gurram S., Barbosa-Cánovas G.V. (2007). High hydrostatic pressure processing on microstructure of probiotic low-fat yogurt. Food Res. Int..

[14] Najafi N.M., Koocheki A., Rezaii Z. Investigation of the effect of whey protein concentration on the properties of soft frozen yogurt. Proceedings of the 9th International Hydrocolloids Conference.

[15] Ghalem B.R., Zouaoui B. (2013). Microbiological, physico-chemical and sensory quality aspects of yogurt enriched with *Rosmarinus officinalis* oil. Afr. J. BioTechnol..

[16] Oluwatuyi M., Kaatz G.W., Gibbons S. (2004). Antibacterial and resistance modifying activity of Rosmarinus officinalis. Phytochemistry.

[17] Mishra A.P., Devkota H.P., Nigam M., Adetunji C.O., Srivastava N., Saklani S., Shukla I., Azmi L., Shariati M.A., Coutinho H.D.M. (2020). Combination of essential oils in dairy products: A review of their functions and potential benefits. LWT-Food Sci. Technol..

[18] Sadler G.D., Murphy P.A., Nielsen S.S. (2010). pH and Titratable Acidity. Food Analysis.

[19] AOAC Association of Official Analytical Chemists (2000). Official Methods 965.33. Official Methods of Analysis.

[20] Kleyn D.H., Lynch J.M., Barbano D.M., Bloom M.J., Mitchell M.W., Cooper L.S., Cusak E., Fick M., Hanks T., Hesen M.K. (2001). Determination of Fat in Raw and Processed Milks by the Gerber Method: Collaborative Study. J. AOAC Int..

[21] Singleton V.L., Orthofer R., Lamuela-Raventós R.M., Lester P. (1999). Analysis of total phenols and other oxidation substrates and antioxidants by means of Folin-Ciocalteu reagent. Methods in Enzymology.

[22] Marinova D., Ribarova F., Atanassova M. (2005). Total phenolics and total flavonoids in Bulgarian fruits and vegetables. J. Univ. Chem. Technol. Metallur..

[23] Wehr H.M., Frank J.F. (2004). Standard Methods for the Examination of Dairy Products.

[24] De Man J.C., Rogosa M., Sharpe M.E. (1960). A Medium for the Cultivation of Lactobacilli. J. Appl. Bacteriol..

[25] Ashenafi M. (1990). Microbiological quality of ayib, a traditional Ethiopian cottage cheese. Int. J. Food Microbiol..

[26] Rashad Y.M.G., Faid S.M. (2014). Effect of using different types of yeasts on the quality of Egyptian balady bread. J. Am. Sci..

[27] Kamel D.G., Hammam A.R.A., Khalid A., Dina A., Osman M. (2020). Addition of inulin to probiotic yogurt: Viability of probiotic bacteria (Bifidobacterium bifidum) and sensory characteristics. Food Sci. Nutr..

[28] Adris A.A., Tower M.A., Soultan A.A.A., Bellail A.A., Ibrahim F.A.A. (2019). Antioxidant and Antimicrobial Activities of *Rosmarinus officinalis* L. Growing Naturally in El-Jabal El-Akhdar Province –Libya and its Effect on Keeping Quality of Cold Serola dumeriri Fillets. J. Food Dairy Sci. Mansoura Univ..

[29] Olmedo R.H., Nepote V., Grosso N.R. (2013). Preservation of sensory and chemical properties in flavoured cheese prepared with cream cheese base using oregano and rosemary essential oils. LWT-Food Sci. Technol..

[30] Binzet G., Binzet R., Arslan H. (2020). The essential oil compositions of *Rosmarinus officinalis* L. leaves growing in Mersin, Turkey. Eur. J. Chem..

[31] Fecka I., Turek S. (2008). Determination of polyphenolic compounds in commercial herbal drugs and spices from Lamiaceae: Thyme, wild thyme and sweet marjoram by chromatographic techniques. Food Chem..

[32] Elena P.C., Rocio J., Julio E.P., Manuel A., Javier V. (2009). Antioxidant activity of seed polyphenols in fifteen wild Lathyrus species from South Spain. LWT-Food Sci. Technol..

[33] Arts I.C., Hollman P.C. (2005). Polyphenols and disease risk in epidemiologic studies. Am. J. Clin. Nutr..

[34] Kholy W.E., Aamer R.A., Mailam M.A. (2017). Effect of Some Essential Oils on the Quality of UF-Soft Cheese During Storage. Alex. J. Food Sci. Technol..

[35] Jardak M., Elloumi-Mseddi J., Aifa S., Mnif S. (2017). Chemical composition, anti-biofilm activity and potential cytotoxic effect on cancer cells of *Rosmarinus officinalis* L. essential oil from Tunisia. Lipids Health Dis..

[36] Fu Y., Zu Y., Chen L., Shi X., Wang Z., Sun S., Efferth T. (2007). Antimicrobial activity of clove and rosemary essential oils alone and in combination. Phytother. Res..

[37] Taheri M., Monshizadeh M., Kordiani H.E. (2015). The relationship between organizational culture and organizational success: A case study. Manag. Sci. Lett..

[38] Hamedo H.A. (2009). Monitoring of Antimicrobial Activity of Essential Oils Using Molecular Markers. Open Biotechnol. J..

[39] Bousbia N., Vian M.A., Ferhat M.A., Petitcolas E., Meklati B.Y., Chemat F. (2009). Comparison of two isolation methods for essential oil from rosemary leaves: Hydrodistillation and microwave hydro diffusion and gravity. Food Chem..

[40] Li L., Li Z.W., Yin Z.Q., Wei Q., Jia R.Y., Zhou L.J., Xu J., Song X., Zhou Y., Du Y.H. (2014). Antibacterial activity of leaf essential oil and its constituents from Cinnamomum longepaniculatum. Int. J. Clin. Exp. Med..

[41] Yeddes W., Nowacka M., Rybak K., Younes I., Hammami M., Saidani-Tounsi M., Witrowa-Rajchert D. (2019). Evaluation of the Antioxidant and Antimicrobial Activity of Rosemary Essential Oils as Gelatin Edible Film Component. Food Sci. Technol. Res..

[42] Valková V., Dúranová H., Galovicová L., Vukovic N.L., Vukic M., Kacániová M. (2021). In Vitro Antimicrobial Activity of Lavender, Mint, and Rosemary Essential Oils and the Effect of Their Vapours on Growth of *Penicillium* spp. In a Bread Model System. Molecules.

[43] Moreira M.R., Ponce A.G., del Valle C.E., Roura S.I. (2005). Inhibitory parameters of essential oils to reduce a foodborne pathogen. Lebens-Mittel-Wiss. Und-Technol.-LWT.

[44] Abers M., Schroeder S., Goelz L., Sulser A., St. Rose T., Puchalski K., Langland J. (2021). Antimicrobial activity of the volatile substances from essential oils. BMC Complement. Med. Ther..

[45] Matsuzaki Y., Tsujisawa T., Nishihara T., Nakamura M., Kakinoki Y. (2013). Antifungal activity of chemo type essential oils from rosemary against Candida albicans. Open J. Stomatol..

[46] Kabouche Z., Boutaghane N., Laggoune S., Kabouche A., Ait-Kaki Z., Benlabed K. (2005). Comparative antibacterial activity of five Lamiaceae essential oils from Algeria. Int. J. Aromather..

[47] Zakia L.L. (1988). Spices and herbs—Their antimicrobial activity and its determination. J. Food Saf..

[48] Deans S.G., Noble R.C., Hiltunen R., Wuryani W., Penzes L.G. (1995). Antimicrobial and antioxidant properties of *Syzygium aromaticum* (L.) Merr. & Perry impact upon bacteria, fungi and fatty acid levels in ageing mice. Flav. Frag. J..

[49] Al-Marzoqi A.H., Hameed I.H., Idan S.A. (2015). Analysis of bioactive chemical components of two medicinal plants (Coriandrum sativum and Melia azedarach) leaves using gas chromatography-mass spectrometry (GC-MS). Afr. J. BioTechnol..

[50] Udaweediye L.R., Premathilaka R., Ginigandarage M.S., Wiranjanee S. (2014). Bioactive compounds and antioxidant activity of bunchosia armeniaca. World J. Pharm. Pharm. Sci..

[51] Saeidnia S., Wexler P. (2014). Phthalate. Encyclopedia of Toxicology.

[52] Karthi S., Somanath B., Ali A.H. (2015). Efficacy of Methanolic Extract of a Marine Ascidian, Lissoclinum bistratum for Antimicrobial Activity. J. Chem. Biol. Phys. Sci..

[53] Vallianou I., Hadzopoulou-Cladaras M. (2016). Camphene, a Plant Derived Monoterpene, Exerts Its Hypolipidemic Action by Affecting SREBP-1 and MTP Expression. PLoS ONE.

[54] Erland L., Bitcon C.R., Lemke A.D., Mahmoud S.S. (2016). Antifungal Screening of Lavender Essential oils and Essential Oil Constituents on three Post-harvest Fungal Pathogens. Nat. Prod. Commun..

[55] Van der Wat L., Dovey M., Naudé Y., Forbes P.B.C. (2013). Investigation into the Aroma of Rosemary using Multi-Channel Silicone Rubber Traps, Off-line Olfactometry and Comprehensive Two-dimensional Gas Chromatography-Mass SpectrometryS. Afr. J. Chem..

[56] Sun J. (2007). D-Limonene: Safety and clinical applications. Altern. Med. Rev..

[57] Zuccarini P., Soldan G. (2009). Camphor: Benefits and risks of a widely used natural product. Acta Biol. Szeged..

[58] Chisholm M.G., Wilson M.A., Gaskey G.M. (2003). Characterization of aroma volatiles in key lime essential oils (Citrus aurantifolia Swingle). Flavour Fragr. J..

[59] Priestap H.A., van Baren C.M., Lira P.D.L., Coussio J.D., Bandoni A.L. (2003). Volatile constituents of Aristolochia argentina. Phytochemistry.

[60] Sujatha P., Evanjaline M., Muthukumarasamy S., Mohan V.R. (2017). Determination of bioactive components of Barleria Courtallica Nees (ACANTHACEAE) by gas chromatography-mass spectrometry analysis. Asian J. Pharm. Clin. Res..

[61] Türkez H., Çelik K., Toğar B. (2014). Effects of copaene, a tricyclic sesquiterpene, on human lymphocytes cells in vitro. Cytotechnology.

[62] Oruch R., Pryme I.F. (2012). The biological significance of vitamin A in humans: A review of nutritional aspects and Clinical considerations. Sci. Jet.

[63] Pavithra P.S., Amr S.V. (2018). Aromadendrene oxide 2, induces apoptosis in skin epidermoid cancer cells through ROS mediated mitochondrial pathway. Life Sci..

[64] Altameme H.J., Hameed I.H., Abdulhasan K.M. (2015). Analysis of alkaloid phytochemical compounds in the ethanolic extract of Datura stramonium and evaluation of antimicrobial activity. Afr. J. Biotechnol..

[65] Hadi M.Y., Mohammed G.J., Hameed I.H. (2016). Analysis of bioactive chemical compounds of Nigella sativa using gas chromatography-mass spectrometry. J. Pharmacogn. Phytother..

[66] Mumtaz M.Z., Kausar F., Hassan M., Javaid S., Malik A. (2021). Anticancer activities of phenolic compounds from Moringa oleifera leaves: In vitro and in silico mechanistic study. Beni-Suef Univ. J. Basic Appl. Sci..

[67] Parker J.K., Parker J.K., Elmore S., Methven L. (2015). Introduction to Aroma Compounds in Foods. Flavour Development, Analysis and Perception in Food and Beverages.

[68] Al Bratty M., Makeen H.A., Alhazmi H.A., Syame S.M., Abdalla A.N., Homeida H.E., Sultana S., Ahsan W., Khalid A. (2020). Phytochemical, Cytotoxic, and Antimicrobial Evaluation of the Fruits of Miswak Plant, *Salvadora persica* L.. J. Chem..

[69] Kadhim W.A., Kadhim M.J., Hameed I.H. (2017). Antibacterial Activity of Several Plant Extracts Against Proteus Species. Int. J. Pharm. Clin. Res..

[70] Dan T., Wang D., Jin R.L., Zhang H.P., Zhou T.T., Sun T.S. (2017). Characterization of volatile compounds in fermented milk using solid-phase micro extraction methods coupled with gas chromatography-mass spectrometry. J. Dairy Sci..

[71] Hassan W.H., El Gamal A.A., El-Sheddy E., Al-Oquil M., Farshori N.N. (2014). The chemical composition and antimicrobial activity of the essential oil of Lavandula coronopifolia growing in Saudi Arabia. J. Chem. Pharm. Res..

[72] Lee H.S., Bilehal D., Lee G.S., Ryu D.S., Kim H.K., Suk D.H., Lee D.S. (2013). Anti-inflammatory effect of the hexane fraction from Orostachys japonicusin RAW 264.7 cells by suppression of NF-κB and PI3K-Akt signaling. J. Funct. Foods.

[73] Hussein J., Hussein M., Hadi Y., Imad I.H. (2016). Study of chemical composition of Foeniculum vulgare using Fourier transform infrared spectrophotometer and gas chromatography—Mass spectrometry. J. Pharmacogn. Phytother..

[74] Guler M.O., Stupp S.I. (2007). A Self-Assembled Nanofiber Catalyst for Ester Hydrolysis. J. Am. Chem. Soc..

[75] Gurbuz B., Bahtiyarca R., Uyanik M., Rezaeieh K.A.P. (2016). Rosemary (*Rosmarinus officinalis* L.) cultivation studies under Ankara ecological coditions. Ind. Crop. Prod..

[76] Senthil J., Rameashkannan M.V., Mani P. (2016). Phytochemical profiling of ethanolic leaves extract of lpomoea sepiara (Koenig Ex. Roxb). IJIRSET.

[77] Ali H.I., Dey M., Alzubaidi A.K., Alneamah S.J.A., Altemimi A.B., Pratap-Singh A. (2021). Effect of Rosemary (*Rosmarinus officinalis* L.) Supplementation on Probiotic Yogurt: Physicochemical Properties, Microbial Content, and Sensory Attributes. Foods.

[78] Zhong J., Yang R.X., Cao X., Liu X., Qin X. (2018). Improved Physicochemical Properties of Yogurt Fortified with Fish Oil/γ-Oryzanol by Nanoemulsion Technology. Molecules.

[79] Abdalla O.M., Abdel Nabi A.S.Z. (2010). Chemical Composition of Mish “A Traditional Fermented Dairy Product” from Different Plants during Storage. Pak. J. Nutr..

[80] Al-Soudy M., E-Batawy O.I., Abdel Fattah A.A., Safaa T.G., El-Dsouky W.I. (2000). Production of function yoghurt drink fortified with different types of herbal extracts and its biological attributes in hepatitis rats. Arab Univ. J. Agric. Sci..

[81] El-Sayed S.A., Mahmood S.S., Mohamed A.F., Ahmed E., Mohamed A.E. (2018). Chemical Composition of Hydro distillation and Solvent Free Microwave Extraction of Essential Oils from *Mentha piperita* L. Growing in Taif, Kingdom of Saudi Arabia, and their Anticancer and Antimicrobial Activity. Orient. J. Chem..

[82] Thabet H.M., Nogaim Q.A., Qasha A.S., Abdoalaziz O., Alnsheme N. (2014). Evaluation of the effects of some plant derived essential oils on shelf-life extension of Labneh. Merit Res. J. Food Sci. Technol..

[83] Okoh O.O., Sadimenko A.P., Afolayan A.J. (2010). Comparative evaluation of the antibacterial activities of the essential oils of *Rosmarinus officinalis* L. obtained by hydrodistillation and solvent free microwave extraction methods. Food Chem..

[84] Kaminarides S., Stamou P., Massouras T. (2007). Comparison of the Characteristics of Set Type Yogurt Made from Ovine Milk of Different Fat Content. Int. J. Food Sci. Technol..

